# TCR-epiDiff: solving dual challenges of TCR generation and binding prediction

**DOI:** 10.1093/bioinformatics/btaf202

**Published:** 2025-07-15

**Authors:** Se Yeon Seo, Je-Keun Rhee

**Affiliations:** Department of Bioinformatics & Life Science, Soongsil University, Seoul 06978, Korea; Department of Bioinformatics & Life Science, Soongsil University, Seoul 06978, Korea

## Abstract

**Motivation:**

T cell receptors (TCRs) are fundamental components of the adaptive immune system, recognizing specific antigens for targeted immune responses. Understanding their sequence patterns is crucial for designing effective vaccines and immunotherapies. However, the vast diversity of TCR sequences and complex binding mechanisms pose significant challenges in generating TCRs that are specific to a particular epitope.

**Results:**

Here, we propose TCR-epiDiff, a diffusion-based deep learning model for generating epitope-specific TCRs and predicting TCR–epitope binding. TCR-epiDiff integrates epitope information during TCR sequence embedding using ProtT5-XL and employs a denoising diffusion probabilistic model for sequence generation. Using external validation datasets, we demonstrate the ability to generate biologically plausible, epitope-specific TCRs. Furthermore, we leverage the model’s encoder to develop a TCR–epitope binding predictor that shows robust performance on the external validation data. Our approach provides a comprehensive solution for both *de novo* generation of epitope-specific TCRs and TCR–epitope binding prediction. This capability provides valuable insights into immune diversity and has the potential to advance targeted immunotherapies.

**Availability and implementation:**

The data and source codes for our experiments are available at: https://github.com/seoseyeon/TCR-epiDiff.

## 1 Introduction

T cells play a crucial role in recognizing diverse antigens derived from pathogens, tumors, and the environment while maintaining immune memory and self-tolerance (Kumar *et al.* 2018, Belk *et al.* 2022). The T cell receptor (TCR) specifically binds to short peptides presented by major histocompatibility complex (MHC) molecules, initiating a robust immune response. TCRs comprise four distinct polypeptides (α, β, γ, δ) forming two heterodimers (αβ and γδ), which share structural, genetic, and functional similarities with immunoglobulins in terms of primary sequence, gene composition, and rearrangement mechanisms (Davis and Bjorkman 1988). Effective T cell activation requires additional ligand–receptor interactions, often referred to as co-stimulatory signals (Marrack and Kappler 1987, Dunn *et al.* 2019), representing highly regulated mechanism optimized for specific antigen recognition (Krummel *et al.* 2016). The diversity and specificity of TCR sequences enable the immune system to accurately target a wide array of antigens.

The precise antigen recognition capability of TCRs has led to their exploitation in T cell-based therapeutic approaches. Notably, chimeric antigen receptors (CARs) are engineered receptors capable of conferring arbitrary specificity to T cells (Shah and Fry 2019). CAR-T cells can identify specific tumor antigens, thereby detecting and eradicating cancer cells. Since 2017, the US Food and Drug Administration (FDA) has approved several CAR-T cell therapies for the treatment of hematologic malignancies (Chen *et al.* 2023). However, the efficacy of CAR-T therapies remains limited in solid tumors. To address this limitation, TCR-T cell therapies have been developed, specifically designed to target solid tumors, which constitute approximately 95% of all cancers (Zheng *et al.* 2022, Zhao *et al.* 2024). Unlike CAR-T cells, TCR-T cells can recognize neoantigens, making them particularly suited for solid tumor therapy (Liu *et al.* 2021). Despite the potential, identifying TCRs that are both antigen-specific and self-tolerant, capable of eradicating established tumors and preventing recurrence, remains a significant challenge.

Recently, the expanded scale and inherent complexity of biological data have driven the adaptation of machine learning approaches (Greener *et al.* 2022). In particular, various deep learning architectures such as convolutional neural networks (CNNs), long short-term memory (LSTM) networks, and generative adversarial networks (GANs) have been successfully applied to bioinformatics challenges, including protein function prediction, protein–ligand interaction prediction, and gene expression prediction (Sapoval *et al.* 2022). Furthermore, notably, Denoising Diffusion Probabilistic Models (DDPMs) have demonstrated superior performance in areas such as image generation, image inpainting, and speech synthesis (Guo *et al.* 2024), and have been also leveraged to generate biological data (Ho *et al.* 2020, Shi *et al.* 2024, Zhou *et al.* 2024a). DDPMs operate by gradually adding Gaussian noise to input data during the forward process, until the original pattern becomes no longer distinguishable. Then, in the reverse process, the noise is sequentially removed to reconstruct the original data.

**Figure 1.**
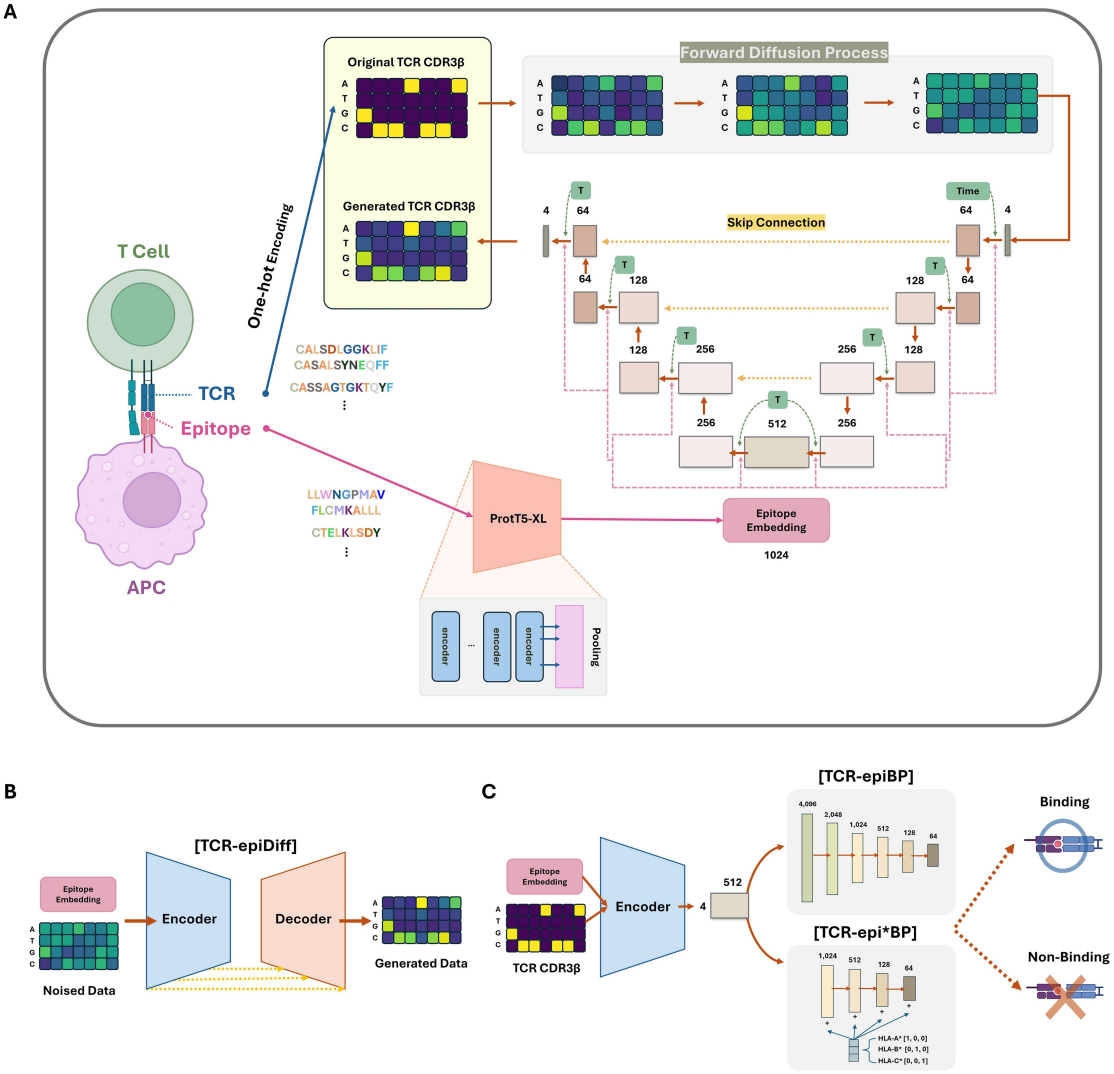
Overview of the TCR-epiDiff architecture. (A) Schematic illustration of TCR-epiDiff architecture. (B) Illustration of the generative process. (C) Structure of the TCR–epitope binding prediction model. The figure was created with BioRender.com.

Here, we propose TCR-epiDiff, a DDPM-based model for generating epitope-specific TCRs (Fig. 1). The model incorporates epitope information at each layer of the model enabling the *de novo* generation of TCRs specific to target epitopes (Fig. 1A and B). We validated our approaches using COVID-19 datasets and confirmed the successful generate of novel TCRs specific to epitope. Furthermore, we utilized the TCR-epiDiff encoder to build a classifier for TCR–epitope binding prediction (Fig. 1C). It also achieved robust performance on the external validation data, highlighting the ability to predict TCR–epitope binding using only sequence data. This dual capability of TCR generation and binding prediction would provide new insights for developing targeted immunotherapies and precision cancer treatments.

## 2 Materials and methods

### 2.1 Data preparation

We utilized three databases for model training ($S^{+}$): VDJdb (Shugay *et al.* 2018, Bagaev *et al.* 2020), McPas (Tickotsky *et al.* 2017), and Gliph (Huang *et al.* 2020), to collect CDR3β sequences and their corresponding binding epitope sequences. We selected CDR3β sequences with amino acid lengths between 7 and 24, following the previous studies (Leary *et al.* 2024). As a result, we obtained 50 310 CDR3β sequences and 1348 epitope sequences. Subsequently, each amino acid was converted into nucleic acid sequences using the most frequently observed human codons. For inputting the sequence data into the DDPM, we applied one-hot encoding, where nucleotides were encoded as follows: A = [1, 0, 0, 0], T = [0, 1, 0, 0], G = [0, 0, 1, 0], and C = [0, 0, 0, 1]. For training, the dataset was equally split between TCR generation and binding prediction models. That is, 50% of the total dataset was used to train the TCR generation model, while the remaining 50% was used for the TCR–epitope binding prediction model.

Negative samples for TCR–epitope binding were generated by randomly sampling from a pool of healthy PBMC TCRs for each peptide (Gao *et al.* 2023), obtained from donor 1 (https://www.10xgenomics.com/cn/datasets/cd-8-plus-t-cells-of-healthy-donor-1-1-standard-3-0-2). The negative sample pairs were constructed by matching the healthy PBMC TCRs with the randomly selected epitopes ($S_{\mathrm{RANDOM}}^{-}$). Additionally, we complied new negative epitopes from the Immune Epitope Database (IEDB), which provides experimental identification results. The IEDB-based new negative samples were constructed by random pairing these negative epitopes with the healthy PBMC TCRs from donor 1 ($S_{\mathrm{IEDB}}^{-}$).

As a result, we compiled a training dataset comprising 25 148 positive and 25 000 negative samples ($S_{\mathrm{RANDOM}}^{-}$) for TCR-epiBP. Furthermore, we constructed another model to predict TCR–epitope binding that incorporates HLA typing information (TCR-epi*BP). For TCR-epi*BP, comprising 23 633 positive samples with corresponding HLA-type annotations from the VDJdb and 25 000 IEDB-based negative samples ($S_{\mathrm{IEDB}}^{-}$).

For external validation, we used a COVID-19 dataset comprising 8496 CDR3β sequences and 633 epitope sequences (Bagaev *et al.* 2020) and a NeoTCR (Zhou *et al.* 2024b) dataset comprising 132 CDR3β sequences and 50 epitope sequences. In both datasets, only the entries with HLA typing information were extracted. Although the COVID-19 data were also sourced from VDJdb, they were not included in the training samples. The negative datasets for the two external validation datasets were processed in the same manner as the training data, but the healthy PBMC CDR3β sequences were randomly retrieved from healthy donor 2 (https://www.10xgenomics.com/cn/datasets/cd-8-plus-t-cells-of-healthy-donor-2-1-standard-3-0-2). Consequently, the COVID-19 dataset consists of 8496 positive and 8496 IEDB-based negative samples, while the NeoTCR dataset consists of 132 positive and 116 IEDB-based negative samples.

### 2.2 Model architecture

TCR-epiDiff was inspired by DDPM. The model employs a U-Net architecture that projects the encoded sequences into 512 dimensions. The down sampling stage consists of convolutional layers that progressively reduce the spatial dimensions until the desired channel dimension is reached. The up sampling stage mirrors the down sampling stage, except that the down sampling convolutional layers are replaced with up sampling convolutional layers. Consequently, the U-Net produces an output with the same dimensionality as the input.

A key feature of the architecture is the use of skip connections between the down sampling and up sampling stages. These skip connections utilize the residual learning technique, which helps preserve important features from earlier layers and mitigates the vanishing gradient problem, thereby improving the overall training stability and model performance.

Additionally, we encoded the diffusion time step (*t*) using sinusoidal embeddings (Vaswani *et al.* 2017), followed by a small multilayer perceptron. The resulting timestep embedding is concatenated with the input of each denoising block to condition the model on the current diffusion stage. To generate epitope-specific TCRs, we integrated epitope information into each layer of the DDPM. The epitope sequences were embedded using proT5-XL, a pre-trained protein language model that captures contextual protein properties (Pokharel *et al.* 2022). These embeddings, with a dimension of (1, 1024), were concatenated into every layer of the TCR-epiDiff model to ensure the epitope-specificity of the generated TCRs. Epitope encodings obtained from proT5-XL are also adjusted to match the dimensionality of each layer, allowing them to be seamlessly integrated throughout the model.

For the TCR–epitope binding prediction, we constructed two models; TCR-epiBP and TCR-epi*BP. While TCR-epiBP was trained using the data from conventional negative sampling ($S_{\mathrm{RANDOM}}^{-}$) without considering HLA types, TCR-epi*BP incorporated the HLA types and was trained with the IEDB-based negative samples ($S_{\mathrm{IEDB}}^{-}$) to account for the influence of peptide–MHC interactions on TCR recognition. These models were developed by extending the TCR-epiDiff model by adding seven (for TCR-epiBP) and five (for TCR-epi*BP) linear layers after the encoder (down sampling stage), to create a model for predicting TCR–epitope binding. The HLA types were converted into one-hot encodings as follows: HLA-A* = [1, 0, 0], HLA-B* = [0, 1, 0], and HLA-C* = [0, 0, 1]. This information was then concatenated with each layer. Dropout (0.6) was applied to all layers except for the final layer.

### 2.3 Training process

The training process consists of forward and reverse processes, following the diffusion model frameworks. During the forward process, Gaussian noise is gradually added to the input data over a series of time steps. The transition probability from $X_{t-1}$ to $X_{t}$ at time step *t* is defined as follows:


$$q(X_{t}|X_{t-1}):=N(X_{t};\sqrt{1-\beta_{t}}X_{t-1},\beta_{t}I),$$

which can be expressed as:


$$X_{t}=\sqrt{1-\beta_{t}}X_{t-1}+\sqrt{\beta_{t}}\epsilon_{t-1},\;\epsilon_{t-1}∼N(0,I).$$

$X_{t}$ represents the data state at the current time step *t*, while $X_{t-1}$ corresponds to the data state at the previous time step t−1. $\beta_{t}$ denotes the noise variance at time *t*, and *I* is the identity matrix, used as the covariance matrix of the noise distribution, ensuring the noise follows an independent and identically distributed Gaussian distribution. ϵ represents the noise sampled from a standard normal distribution N(0,I), which contributes to progressively adding noise to the data as it transitions from $X_{t-1}$ to $X_{t}$. A linear noise schedule was used with a $\beta_{\mathrm{start}}$ = 0.0001 and a $\beta_{\mathrm{end}}$ = 0.1. The reverse process learns to denoise the data by minimizing the mean squared error (MSE) between the added noise and the model’s predicted noise as follows:


$$L(\theta )=E_{X_{0},\epsilon }\left[{\left\|\epsilon -\epsilon_{\theta }(X_{t},t)\right\|}^{2}\right],$$

where $X_{t}$ is given by:


$$X_{t}=\sqrt{{\tilde{\alpha }}_{t}}X_{0}+\sqrt{1-{\tilde{\alpha }}_{t}}\epsilon$$

and the terms are defined as:


$$\alpha_{t}:=1-\beta_{t},\;{\tilde{\alpha }}_{t}:=\prod_{s=1}^{t}\alpha_{s}.$$

Here, ${\tilde{\alpha }}_{t}$ represents the cumulative product of $\alpha_{s}$ up to time step *t*, θ denotes the model parameters, and $\epsilon_{\theta }$ is the noise predicted by the model. $E_{X_{0},\epsilon }$ denotes the expected value over the initial data distribution and noise. This loss function guides the model to learn the underlying data distribution through the iterative reverse process, during which noise is progressively removed to recover the original data.

The model was implemented using PyTorch (v2.4.1) and trained on an NVIDIA GeForce RTX 2080 Ti GPU. The Adam optimizer was employed with a learning rate of 1e−4.

### 2.4 Model evaluation

To assess the similarity between the generated TCR sequences and the original sequences obtained from TCR-epiDiff, we calculated the Pearson correlation at each position of the sequences using SciPy (v1.10.1). Additionally, we computed the cosine similarity between the generated CDR3β sequences and the original CDR3β sequences. Negative samples collected earlier were also included in the analysis to enable comparisons. We verified whether TCRs targeting the same epitope are positioned closely in the latent space of the model’s encoder. We reduced the latent TCRs to 10 components using principal component analysis (PCA) in the scikit-learn package (v1.3.2). Subsequently, we applied UMAP with the hyperparameters (n_neighbors = 15, min_dist = 0.3, n_components = 2, metric = “hamming”, spread = 0.5) to visualize the data. The epitopes associated with 45–60 TCRs were selected for the visualization. Nucleic acid and amino acid sequences were visualized using Logomaker (v0.8).

To evaluate the performances and the robustness of our binding prediction models, we compared our two models against four machine learning-based models; NetTCR-2.0 (Montemurro *et al.* 2021), pMTnet (Lu *et al.* 2021), epiTCR (Pham *et al.* 2023), and ERGO-II (Springer *et al.* 2021). NetTCR-2.0 uses CNN model, while pMTnet and ERGO-II utilize LSTM networks, and epiTCR uses a random forest for model construction. The external validation was conducted using the COVID-19 dataset and NeoTCR dataset.

## 3 Results

In this study, we address two critical challenges simultaneously. The first objective was to generate TCR sequences specific to a given epitope, and the second objective was to enable the prediction of TCR–epitope binding.

### 3.1 Generation of epitope-specific TCRs

**Figure 2.**
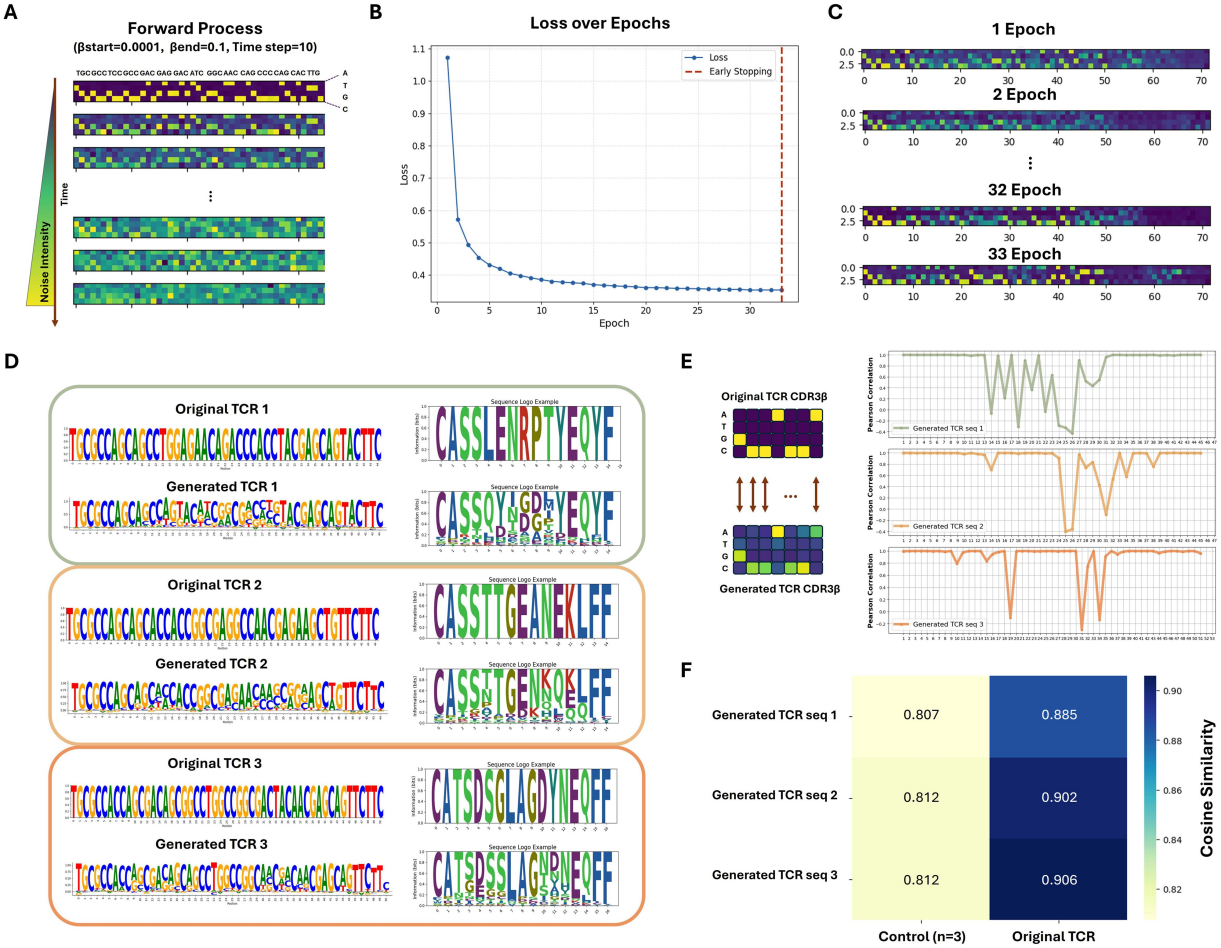
Epitope-specific TCR sequence generation with TCR-epiDiff. (A) Visualization of the forward process during training. The progression shows the gradual addition of Gaussian noise across 10 timesteps. The one-hot encoded initial sequence is composed of 0 (purple) and 1 (yellow). (B) Learning curve over epochs. The dashed red line indicates the early stopping point. (C) Sequence reconstruction at different training stages. The progression from epoch 1 to 33 demonstrates the model’s improving ability to denoise and reconstruct TCR sequences. (D) Comparison between original and generated TCR sequences. Three pairs of sequences are shown in both nucleotide (left) and amino acid (right) representations, illustrating the model’s ability to generate sequences that maintain biological characteristics of TCR CDR3β regions. (E) Position-wise correlation analysis between original and generated TCR sequences. (F) Heatmap of cosine similarity to compare the generated sequences with negative control and original TCRs.

We first evaluated whether TCR-epiDiff was effectively trained to generate epitope-specific TCRs. During the training process, Gaussian noise was added linearly across 10 timesteps, with the $\beta_{\mathrm{start}}$ set to 0.0001 and the $\beta_{\mathrm{end}}$ set to 0.1. The initial sequence data consists of 0 (purple) and 1 (yellow), but after the addition of noise, the original values become indistinguishable (Fig. 2A). The model demonstrated progressive improvement in denoising capability, by decreasing loss values across training epochs (Fig. 2B). We used early stopping to end training if the loss did not improve by at least 0.001 for three consecutive epochs. To further assess the model’s ability to denoise data, we visualized the reconstruction of a TCR sample across epochs (Fig. 2C). In the initial epochs, the model showed imperfect reconstruction of the TCR sequences, but as training progressed, the denoised data closely approximated the original data (Fig. 2C).

Furthermore, we evaluated the model generation capability by investigating the TCR sequences from the final batch. TCR-epiDiff was able to generate diverse nucleotide sequences that translated into a wide range of biologically plausible CDR3β amino acid sequences (Fig. 2D). It has been known that CDR3β has conserved terminal sequences, while the middle region exhibits variability (Friedl-Hajek *et al.* 1998). Our model successfully reflected these properties in the generated sequences. Moreover, we computed the Pearson correlation coefficients between the one-hot encoded original TCR CDR3β and the generated TCR CDR3β at each sequence position. The terminal regions showed high correlation, while the middle regions exhibited appropriate variabilities (Fig. 2E). Consequently, our model could effectively generate CDR3β sequences that align with known biological characteristics. This analysis allowed us to identify conserved residues in CDR3β sequences, which could be considered as some critical features of these sequences.

Next, we evaluated the ability of TCR-epiDiff to generate epitope-specific TCRs using negative control data (Fig. 2F). The negative control dataset consisted of TCRs extracted from normal PBMCs, which were assumed not to bind any epitope. We calculated the average cosine similarity between original TCR sequences and generated TCR sequences as well as between the negative control TCR sequences and generated TCR sequences. The generated TCR sequences showed clearly higher cosine similarity to their corresponding epitope-specific original sequences than to the negative control sequences. A high cosine similarity between the original and generated TCR sequences indicates that the generated TCRs are likely to recognize the same epitope. This demonstrates that TCR-epiDiff accurately generates valid TCR sequences. To further verify the abilities to generate the epitope-specific TCR sequences, we generated random nucleotide sequences and compared the cosine similarities using these sequences. The cosine similarity between random nucleotide sequences and negative controls was 0.875, while similarity with original TCR sequences was substantially lower at 0.654. The cosine similarity between the random sequences and generated TCR sequences by our TCR-epiDiff was 0.712. These results confirmed that our model could generate epitope-specific TCRs while maintaining appropriate sequence diversity.

### 3.2 External validation with COVID-19 and NeoTCR data

**Figure 3.**
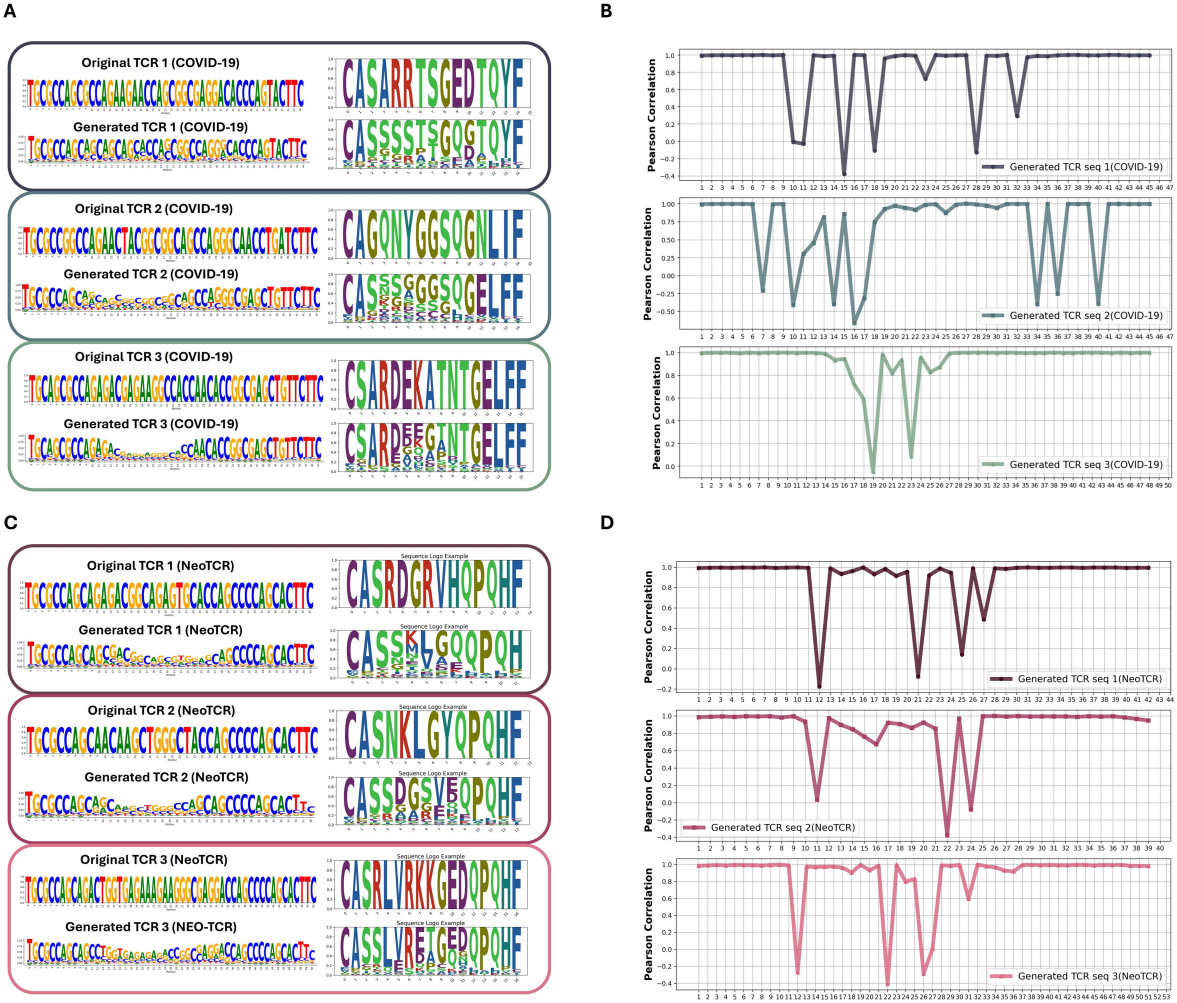
External validation of TCR-epiDiff using COVID-19 and NeoTCR datasets. (A) Comparison of original and generated TCR sequences from COVID-19 dataset. Three representative pairs of sequences are shown in both nucleotide (left) and amino acid (right) formats. (B) Pearson correlation coefficients between original and generated COVID-19 TCR sequences. (C) Comparison of original and generated TCR sequences from NeoTCR dataset. (D) Pearson correlation coefficients between original and generated TCR sequences from NeoTCR dataset.

We investigated the model’s generalization capabilities with COVID-19 and NeoTCR datasets. To verify the model generates the biologically appropriate nucleotide sequences, we carried out the similar evaluation analyses. As we expected, the noise was successfully removed at the nucleotide level in both datasets, leading to the generation of nucleotides with the conserved both terminal sequences (Figs. 3A and C). Furthermore, when these generated nucleotides were translated into amino acids, they resulted in a diverse range of CDR3β amino acid sequences, but the resulting amino acid sequences maintained characteristic CDR3β features, particularly the conservation pattern in terminal regions (Fig. 3B and D).

### 3.3 Latent space analysis for epitope-specific TCRs

**Figure 4.**
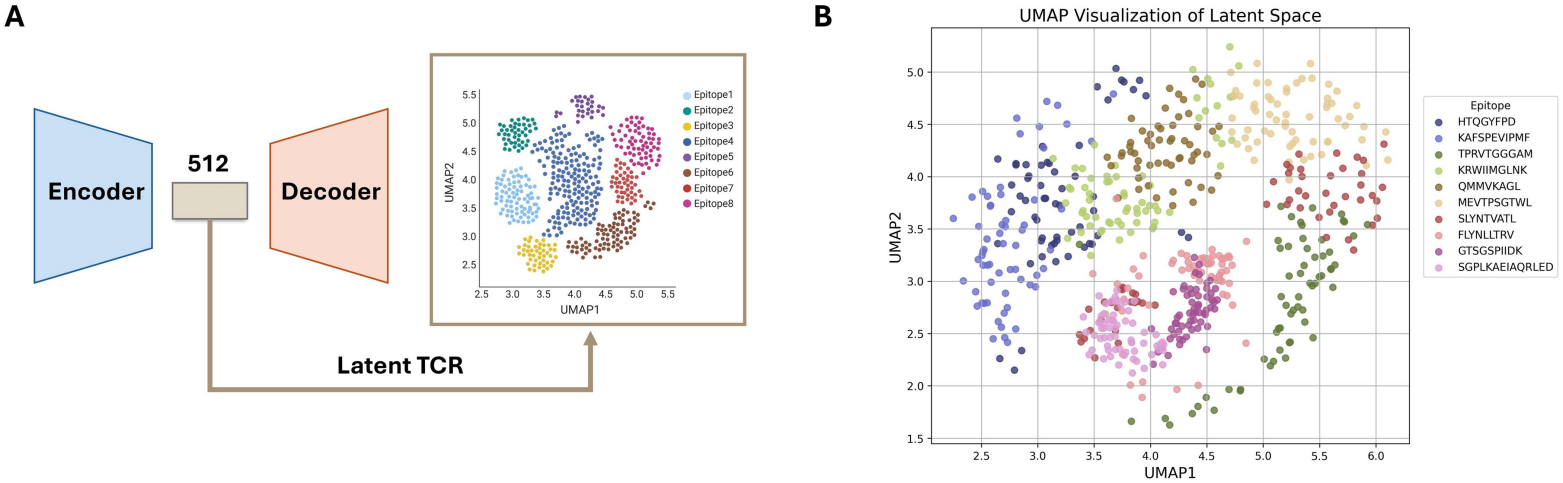
Latent space representation of epitope-specific TCR embeddings. (A) Schematic illustration of the encoder–decoder architecture with 512-dimensional latent space and an example UMAP visualization. (B) UMAP visualization of the latent space representation for TCRs binding to different epitopes. Ten different epitopes are shown, with each point representing a TCR sequence.

Our model is structured in the form of a U-Net, consisting of an encoder and decoder. We evaluated the representation ability of the trained encoder for the effective TCR embedding in an epitope-specific manner. We selected 10 epitopes, each associated with 45–60 CDR3β sequences, and then assessed whether the TCR embedding vectors for these 10 epitopes were clearly separated in the latent space (Fig. 4A). The results demonstrated that our model effectively represents epitope-specific TCRs (Fig. 4B). The TCRs binding with the identical epitopes showed significant spatial proximity in the latent space. For the further exploration, we examined *k*-nearest neighborhood analysis (*k* = 5) in the latent space and found that, for the majority of samples, the neighbors predominantly share the same class (with a proportion of 0.780). This suggests that the model effectively captures the underlying similarity in its internal representation.

### 3.4 TCR–epitope binding prediction

**Figure 5.**
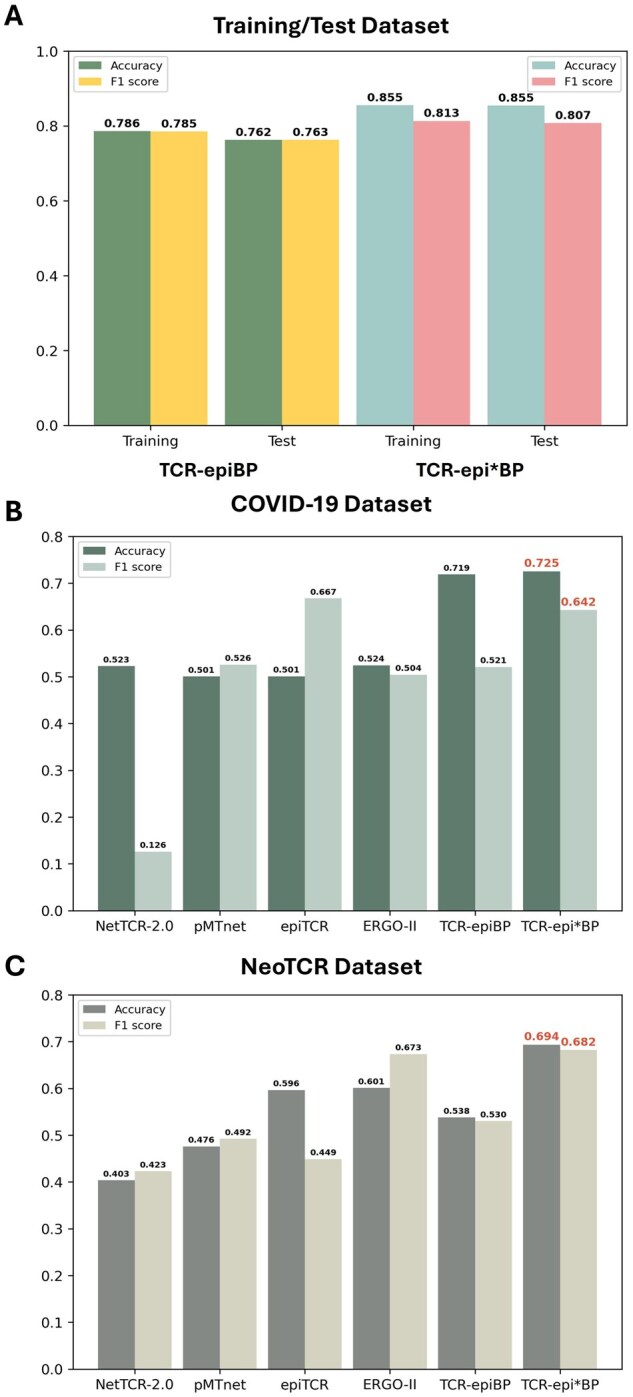
Performance evaluation of the TCR–epitope binding prediction model. (A) Model performance on training and test datasets. (B) External validation results using the COVID-19 dataset. (C) External validation results using the NeoTCR dataset.

Since we confirmed that the encoder effectively represents TCR sequences in an epitope-specific manner, a binding prediction model for CDR3β and epitopes could be developed using the encoder within our model (Fig. 5). The binding prediction model was constructed by adding linear layers to the pre-trained encoder (Fig. 1C). The training performance was evaluated with the hold-out data excluded from the training process of the binding prediction models (around one-fifth of the total samples). For TCR-epiBP, the training accuracy was 0.786 with an *F*1 score of 0.785, and the test accuracy was 0.762 with an *F*1 score of 0.763 (Fig. 5A). For TCR-epi*BP, the training accuracy was 0.855 with an *F*1 score of 0.813, and the test accuracy was 0.855 with an *F*1 score of 0.807.

We also proceeded to perform the classification using the external validation datasets. Using COVID-19 dataset, the evaluation results showed an accuracy of 0.719 and an *F*1 score of 0.521 for TCR-epiBP and an accuracy of 0.725 and an *F*1 score of 0.642 for TCR-epi*BP (Fig. 5B). We further compared our methods with four recent machine learning-based TCR–epitope binding prediction methods. Our two models, TCR-epiBP and TCR-epi*BP, demonstrated performance that is comparable to or outperforms that of these previous models. To further validate our models, we compiled another validation dataset, NeoTCR. The NeoTCR dataset contains experimentally supported paired information between CDR3β sequences and neoantigen. It primarily includes neoantigens caused by single nucleotide mutations, providing insight into antigen binding in solid tumors. Using the NeoTCR dataset, the evaluation results for TCR-epiBP showed performance comparable to that of other methods, whereas TCR-epi*BP outperformed others, achieving an accuracy 0.694 and *F*1 score 0.682 (Fig. 5C). These results demonstrate that our models provide robust binding predictions based on TCR and epitope sequences.

## 4 Discussion

Recent advancements in deep learning have demonstrated its effectiveness in uncovering patterns, relationships, and mechanisms within vast datasets (Zhang *et al.* 2024). Particularly, denoising diffusion models, a type of generative artificial intelligence, have shown significant promise in fields such as computer vision, natural language processing, and bioinformatics (Guo *et al.* 2024). These models are increasingly being utilized for applications like protein design, drug design, protein–ligand interaction modeling, and the analysis of microscopy image data. For instance, FrameDiff successfully applied diffusion models to generate novel functional protein backbones that are not observed in nature (Yim *et al.* 2023).

In this study, we demonstrate the remarkable capability of our model, TCR-epiDiff, to generate epitope-specific TCR sequences. Notably, our model captures and generates the conserved terminal regions of the CDR3β sequence, indicating that it has accurately learned the patterns of CDR3β. The model also achieved outstanding performance on unseen data, including COVID-19 and NeoTCR datasets, highlighting its potential for broad biological applications. In addition, we extended the encoder component of TCR-epiDiff to develop a predictive model for TCR–epitope binding. This success can be attributed to the model’s ability to embed TCR sequences while incorporating epitope information. Moreover, the model can propose candidate TCR sequences for a given epitope based on their similarity in the learned embedding space, aiding in the identification of likely binders from unlabeled TCR datasets. This capability could prove valuable for experimental follow-up and repertoire analysis.

While our model represents significant progress, certain limitations remain. First, the model’s generative outputs require validation through *in vivo* or *in vitro* binding assays. Second, our training data may be biased due to natural selection processes, as strong immune responses are more likely to be observed and recorded. Nevertheless, our models successfully identified the candidate TCR sequences capable of binding to a specific epitope even though using the validation datasets including COVID-19 and neoantigen datasets. Future work should incorporate synthetic or rare epitope data to enhance generalizability. Additionally, if a sufficiently large dataset containing affinity information between TCR sequences and epitopes is available, our model would be extended to predict TCR sequences with a high binding affinity to a specific epitope, thereby enhancing its applicability in personalized immunotherapy. Furthermore, additional variational models should be explored. For example, we followed the standard DDPM setup by using Gaussian noise in the forward process. While the Gaussian noise is widely used and effective, exploring alternative noise strategies may offer new insights or benefits for the sequence generation tasks. Moreover, we used nucleotide-level TCR representations to preserve codon-level variation, including synonymous mutations and codon usage biases, which may influence TCR expression or selection. This approach may enable the model to learn a more fine-grained and diverse representation of TCR repertoires. In the future, it would be valuable to compare the generative and predictive performance of nucleotide- versus amino acid-based models, or to explore hybrid architectures that incorporate both sequence levels.

In conclusion, TCR-epiDiff effectively generates epitope-specific TCR sequences and predicts TCR–epitope interactions, addressing two critical challenges simultaneously. Our model holds potential for use in therapeutic strategies, including cancer vaccines, precision immunotherapy and T cell engineering, representing a significant milestone in the field.

## Author contributions

Se Yeon Seo: Conceptualization, Data curation, Formal analysis, Investigation, Methodology, Resources, Software, Validation, Visualization, Writing—original draft, Writing—review & editing, Je-Keun Rhee: Conceptualization, Funding acquisition, Investigation, Methodology, Project administration, Resources, Supervision, Visualization, Writing—original draft, Writing—review & editing

Conflict of interest: No competing interest is declared.

## Data Availability

The data and source codes for our experiments are available at https://github.com/seoseyeon/TCR-epiDiff. Our model and dataset can be downloaded from https://doi.org/10.5281/zenodo.15094766 and https://doi.org/10.5281/zenodo.15094767.
